# Altered Resting-State Brain Activity in Schizophrenia and Obsessive-Compulsive Disorder Compared With Non-psychiatric Controls: Commonalities and Distinctions Across Disorders

**DOI:** 10.3389/fpsyt.2021.681701

**Published:** 2021-05-21

**Authors:** Yuyanan Zhang, Jinmin Liao, Qianqian Li, Xiao Zhang, Lijun Liu, Jun Yan, Dai Zhang, Hao Yan, Weihua Yue

**Affiliations:** ^1^Institute of Mental Health, Peking University Sixth Hospital, Beijing, China; ^2^Key Laboratory of Mental Health, Ministry of Health & National Clinical Research Center for Mental Disorders, Peking University, Beijing, China; ^3^PKU-IDG/McGovern Institute for Brain Research, Peking University, Beijing, China; ^4^Research Unit of Diagnosis and Treatment of Mood Cognitive Disorder (2018RU006), Chinese Academy of Medical Sciences, Beijing, China

**Keywords:** schizophrenia, obsessive-compulsive disorder, amplitude of low-frequency fluctuations, resting-state functional connectivity, striatum

## Abstract

**Backgrounds:** Schizophrenia (SCZ) and obsessive-compulsive disorder (OCD) are classified as two chronic psychiatric disorders with high comorbidity rate and shared clinical symptoms. Abnormal spontaneous brain activity within the cortical–striatal neural circuits has been observed in both disorders. However, it is unclear if the common or distinct neural abnormalities underlie the neurobiological substrates in the resting state.

**Methods:** Resting-state fMRI data were collected from 88 patients with SCZ, 58 patients with OCD, and 72 healthy control subjects. First, we examined differences in amplitude of low-frequency fluctuations (ALFF) among three groups. Resting-state functional connectivity (rsFC) analysis with the brain region that showed different ALFF as the seed was then conducted to identify the changes in brain networks. Finally, we examined the correlation between the altered activities and clinical symptoms.

**Results:** Both the patients with SCZ and OCD showed increased ALFF in the right hippocampus and decreased ALFF in the left posterior cingulate cortex (PCC). SCZ patients exhibited increased ALFF in the left caudate [voxel-level family-wise error (FWE) *P* < 0.05] and decreased rsFC between the left caudate and right cerebellum, which correlated with positive symptoms. The left caudate showed increased rsFC with the right thalamus and bilateral supplementary motor complex (SMC) in OCD patients (cluster-level FWE *P* < 0.05).

**Conclusions:** The hippocampus and PCC are common regions presenting abnormal local spontaneous neuronal activities in both SCZ and OCD, while the abnormality of the striatum can reflect the differences. Increased ALFF in the striatum and symptom-related weakened rsFC between the caudate and cerebellum showed SCZ specificity. Enhanced rsFC between the caudate and SMC may be a key characteristic in OCD. Our research shows the similarities and differences between the two diseases from the perspective of resting-state fMRI, which provides clues to understand the disease and find methods for treatment.

## Introduction

The categorical diagnoses according to the phenotypic definitions limit the discovery of a genetic association study in psychiatry (1, 2). The symptoms overlap among disorders, and shared biological features indicate a lack of clear boundary in traditional categorical diagnostic systems (3, 4). The recently proposed Hierarchical Taxonomy of Psychopathology model organizes psychopathology into a hierarchy with traits to address problems of diagnostic heterogeneity, comorbidity, and unreliability [The Hierarchical Taxonomy of Psychopathology (HiTOP): A Quantitative Nosology Based on Consensus of Evidence] (5), while endophenotype studies depend on neuroimaging measures to try to develop quantifiable biomarkers for deeper understanding of pathophysiology across classical diagnostic categories and to promote the presentation of a more comprehensive spectrum of psychiatric disorders (6, 7). Here, we try to find out the local spontaneous brain function activity characteristics in schizophrenia (SCZ) and obsessive-compulsive disorder (OCD), two mental disorders with common genetic factors (8, 9) and structural brain abnormalities (10, 11).

SCZ is characterized by consciousness abnormalities including hallucinations, delusions, disorganized speech, decreased motivation, and cognitive deficits (12), while OCD is identified by recurrent intrusive and unwanted thoughts, which result in distress or anxiety and repetitive behaviors (13). The obsessive thoughts in both OCD and delusional ideas in SCZ involved intrusive, unwanted, and foreign thoughts, which indicated the shared failure in monitoring their own thoughts (14). Meta-analysis showed that the total prevalence rate of OCD in SCZ was as high as 12.3% (15). The diagnosis of OCD also increases the risk of SCZ (16). Patients with both disorders showed deficient response inhibition (17) and internal source-monitoring deficits (14). As for etiology researches, the common features of the two disorders can be partially explained by shared polygenic risk (8) and shared pathways of glutamate, dopamine, and serotonin (9). However, the neurobiological substrates and the etiological relationship underlie that the tight association remains unclear.

Previous studies have reported similarities in intrinsic abnormal functions of OCD and SCZ in fronto-striatal circuits. Dysregulated dopaminergic modulation of striatal function is the basis of models that attempt to explain the mechanism of the symptoms in SCZ (18). The hypoconnectivity between the frontal lobe and dorsal striatum has been observed in individuals with SCZ (19, 20). The striatal hyperdopaminergia might disrupt signaling between the frontal cortex and striatum or drive cortical dopamine dysregulation, which results in cognition impairments (18, 21). Cortico-striato-thalamo-cortical (CSTC) circuits are hypothesized as the core neural circuits that underlie OCD, which engage functionally related regions of the cortex, striatum, and thalamus with a direct (net excitatory) or indirect (net inhibitory) pathway (13). Consistent evidence showed increased activity in the brain regions that form a CSTC loop, and overactivity of the direct pathway is hypothesized as a pathogenesis of OCD (22). Increased habit information in the balance between habitual and goal-directed behavior was associated with hyperactivation of the caudate nucleus (23). Neuroimaging studies also have found abnormal resting-state activity related to fronto-striatal circuits in OCD and SCZ. The amplitude of low-frequency fluctuations (ALFF) represents the magnitude of the regional activity amplitude and reflects the intensity of spontaneous neuronal activity. The brain regions with increased ALFF in patients with SCZ were mainly located in the bilateral striatum, medial temporal lobe, and medial prefrontal lobe (24). In patients with OCD, the values of fractional ALFF (fALFF) and the standardization index of ALFF in the putamen and superior frontal gyrus increased (25). On the other hand, a neuroimaging biomarker for functional striatal abnormalities was demonstrated to successfully distinguish SCZ from OCD (26), which suggested that the function of the striatum might reflect the specificity of SCZ to some extent. Considering the core role of the striatum in the dopamine hypotheses of SCZ and CSTC circuits, which are involved in OCD, we speculated that the abnormal function of the striatum may be the common neuropathological mechanism of SCZ and OCD and moreover a valuable marker for distinguishing them. However, there still lack the explorations of differences between SCZ and OCD in ALFF and resting-state functional connectivity (rsFC).

In this study, we aimed to explore the similarities and abnormalities in the brain intrinsic activity of SCZ patients, OCD patients, and healthy controls (HCs) using resting-state functional magnetic resonance imaging (rs-fMRI). First, we attempted to determine the brain regions showing altered local spontaneous brain activity measured by ALFF in SCZ and OCD compared with HCs, with the hypothesis that brain regions within the cortical–striatal neuronal circuits would be vulnerable. Then, we further compared the seed-based rsFC with the brain region in the above ALFF analysis as seeds in SCZ patients, OCD patients, and HCs. Finally, we tested the association between ALFF value of abnormal brain region and showed common and specific features in SCZ and OCD and clinical symptoms to explore the neurobiological mechanism underlying them.

## Methods and Materials

### Participants

All participants were recruited from either the inpatient or outpatient department of Peking University Sixth Hospital (Beijing, China). Inclusion criteria of all participants included being 18–45 years old; Han Chinese ethnicity; and right-handed. To determine SCZ and OCD diagnoses, patients were assessed using the Structured Clinical Interview for DSM-IV Axis I Disorder, Patient Edition (SCID) by an experienced psychiatrist and should be without other comorbidities in the DSM-IV-TR Axis I Disorders (including depression). For HCs, the non-patient edition of the SCID was used to confirm the absence of mental disorders. Participants were excluded if they had the following: a history of neurological disease, a history of >5-min loss of consciousness, or MRI contraindications. This study was approved by the ethics committee of Peking University Sixth Hospital. Written informed consent was obtained from all participants or legal guardians involved in the study.

The Positive and Negative Syndrome Scale (PANSS), which consists of the positive, negative, and general psychopathology subscales, was used to assess SCZ symptoms for patients with SCZ. The Yale–Brown Obsessive Compulsive Symptom Scale (Y-BOCS), which consists of the obsessive thought and compulsive behavior subscales, was used to measure the obsessive-compulsive symptoms for patients with OCD. The Hamilton Anxiety Scale (HAMA) and 17-item Hamilton Depression Scale (HAMD-17) were also used to assess anxiety and depression for patients with OCD.

### MRI Acquisition

All participants were scanned on a 3.0-T GE scanner (Discovery MR750) at the Center for Neuroimaging, Peking University Sixth Hospital. Before scanning, all participants were instructed to move as little as possible. Foam pads were used to minimize head motion. T1-weighted high-resolution structural images were acquired in a sagittal orientation using an axial 3D fast, spoiled gradient recalled (FSPGR) sequence with the following parameters: repetition time (TR) = 6.66 ms, echo time (TE) = 2.93 ms, field of view (FOV) = 256 × 256 mm^2^, slice thickness/gap = 1.0/0 mm, acquisition voxel size = 1 × 1 × 1 mm^3^, flip angle = 12°, and 192 contiguous sagittal slices. The resting-state functional imaging data were acquired with the following parameters: TR = 2,000 ms, TE = 30 ms, FOV = 220 × 220 mm^2^, matrix = 64 × 64, flip angle = 90°, voxel size = 3.5 × 3.5 × 4.2 mm^3^, 33 slices, and 240 volumes. Before scanning, all participants were instructed to move as little as possible, keep their eyes closed, think of nothing in particular, and avoid falling asleep. After scanning, they were asked whether they fell asleep to reconfirm.

### Resting-State fMRI Preprocessing

Data preprocessing of resting-state fMRI was completed using DPABI (27). The following steps were performed: (1) discarding the first 10 volumes from each participant; (2) slice timing correction; (3) realigning the volumes to the middle volume; (4) coregistration using T1 images and spatial normalization by DARTEL (Diffeomorphic Anatomical Registration Through Exponentiated Lie Algebra); (5) linear regression to remove the effects of linear trends; (6) regressing out nuisance covariate signals including white matter and cerebrospinal fluid; and (7) temporal bandpass filtering (0.01–0.1 Hz). Then, the data were smoothed with a Gaussian filter of 6-mm full width at half maximum (FWHM) to reduce noise and residual differences. The voxel size of the image data after preprocessing is 3 × 3 × 3 mm^3^.

To generate the voxel-wise ALFF maps with z-score for each individual, the images were smoothed after the first four preprocessing steps and followed (5) and (6). The ALFF values were calculated in a voxel-wise way as the averaged squared root of the frequency range of 0.01–0.1 Hz.

In addition, a volume-based framewise displacement (FD) was computed based on their realignment parameters to quantify head motion (28, 29). Any subjects with mean FD Jenkinson > 0.2 were excluded (SCZ: *n* = 6; OCD: *n* = 2; HC: *n* = 0). Finally, a total of 88 patients with SCZ, 58 patients with OCD, and 72 HC subjects were included in the further analyses.

### Resting-State Functional Connectivity Analysis

Brain regions showing significantly different ALFF values between the patients with SCZ and OCD were used as seeds in the following rsFC analysis, which was performed using DPABI v4.4. First, the time series of each voxel within the seed were extracted. Second, the extracted time series of each voxel were averaged to acquire the mean time series of the seed. Third, Pearson's correlation coefficients between the mean time series of the seed and the time series of each voxel within the whole brain were calculated and used to construct each subject's rsFC map. Finally, the rsFC maps were converted into z-score maps by Fisher's z transformation to improve normality. The individual rsFC maps with z values were entered into one-way ANOVA to figure out the differences among three groups. Age, gender, education attainment, and mean FD Jenkinson were entered as covariates. A significant level was set at a cluster-level threshold of *P* < 0.05 family-wise error (FWE) corrected. The *post-hoc* pair-wise comparisons were then performed after extracting the rsFC values, and a value of *P* < 0.05 Bonferroni corrected was considered significant.

### Statistical Analysis

Demographic and clinical differences between the patients with OCD, patients with SCZ, and HCs were compared by using one-way ANOVA or χ^2^ test in IBM SPSS Statistics Desktop 26.

Second-level analyses for resting-state fMRI data were performed by using SPM12 (Wellcome Department of Cognitive Neurology, London, UK). One-way ANOVA was used to compare differences of ALFF among the SCZ patients, OCD patients, and HCs within the gray matter mask of the whole brain in DPABI. Age, gender, education attainment, and mean FD Jenkinson were entered as covariates. A significant level was set at a voxel-level threshold of *P* < 0.05 FWE corrected. The *post-hoc* pair-wise comparisons were then performed after extracting the ALFF values, and a value of *P* < 0.05 Bonferroni corrected was considered significant.

Relationships with symptom severity were examined by extracting ALFF and rsFC values from regions showing group differences and correlating these values with PANSS total scores, PANSS positive symptom scores, PANSS negative symptom scores, and PANSS general psychopathology scores in the SCZ group, and Y-BOCS scores, Y-BOCS obsessive thinking scores, Y-BOCS compulsive behavior scores, HAMA scores, and HAMD-17 scores in the OCD group, with age and gender as covariates. A significant level was set at a threshold of *P* < 0.0125 and *P* < 0.01 with Bonferroni correction (for SCZ: *P* < 0.05/4 = 0.0125; for OCD: *P* < 0.05/5 = 0.01).

## Results

There was no significant difference in gender distribution, but in age [*F*_(2,215)_ = 3.994, *P* = 0.020] and years of education [*F*_(2,215)_ = 29.26, *P* < 0.001]. The SCZ group showed the shortest years of education, and the OCD group was the oldest (see details in Table 1). They were all included as covariates in the following analysis. In the 88 patients with SCZ, eight patients were drug-naïve, and 80 patients received atypical antipsychotics (aripiprazole, amisulpride, olanzapine, risperidone, clozapine, quetiapine, paliperidone, and ziprasidone). The chlorpromazine equivalent dose of the antipsychotics (30) was 442.9 ± 305.7 mg/day. Of the 58 patients with OCD, 18 patients were drug-naïve, and 40 patients were taking one or more antidepressants including selective serotonin reuptake inhibitors (SSRIs) (paroxetine, sertraline, fluoxetine, escitalopram, and fluvoxamine), venlafaxine, mirtazapine, clomipramine, and amitriptyline. Thirteen patients were on combined antipsychotic medication in small doses. The fluoxetine equivalent dose of antidepressants (31, 32) was 46.3 ± 44.4 mg/day.

**Table 1:** Demographics and clinical data of the patients with schizophrenia, patients with obsessive-compulsive disorder, and healthy controls.

**Characteristic**	**SCZ**	**OCD**	**HC**	***F*/χ^**2**^**	***P***
Gender (male/female)	53/35	37/21	34/38	4.270	0.118
Age (years)	25.2 ± 6.4	27.2 ± 6.6	24.4 ± 3.4	3.994	0.020
Education (years)	13.6 ± 2.9	15.1 ± 2.8	16.8 ± 2.1	29.26	<0.001
Framewise displacement	0.068 ± 0.038	0.066 ± 0.038	0.058 ± 0.034	1.428	0.242
Onset age (years)	22.1 ± 6.6	19.6 ± 5.5	-	-	-
Disease course (months)	45.8 ± 52.6	95.2 ± 66.9	-	-	-
PANSS total score	69.26 ± 14.62	-	-	-	-
PANSS positive symptoms	18.90 ± 5.86	-	-	-	-
PANSS negative symptoms	17.02 ± 5.48	-	-	-	-
PANSS general psychopathology	33.34 ± 7.98	-	-	-	-
Y-BOCS total score	-	21.46 ± 7.52^∧^	-	-	-
Y-BOCS obsessive thinking	-	11.28 ± 3.80^∧^	-	-	-
Y-BOCS compulsive behavior		10.18 ± 4.75^∧^			
HAMA	-	11.15 ± 6.89^†^	-	-	
HAMD-17	-	7.98 ± 5.22^†^	-	-	-

*Data are given as mean ± standard deviation. P-values refer to one-way ANOVA (parametric data) and chi-square test (categorical data)*.

*SCZ, patients with schizophrenia; OCD, patients with obsessive-compulsive disorder; HC, healthy controls; PANSS, Positive and Negative Syndrome Scale; Y-BOCS, Yale–Brown Obsessive-Compulsive Scale; HAMA, Hamilton Anxiety Scale; HAMD-17, 17-item Hamilton Depression Scale; -, not applicable*.

∧ *N = 50;*

† *N = 53*.

**Figure 1:**
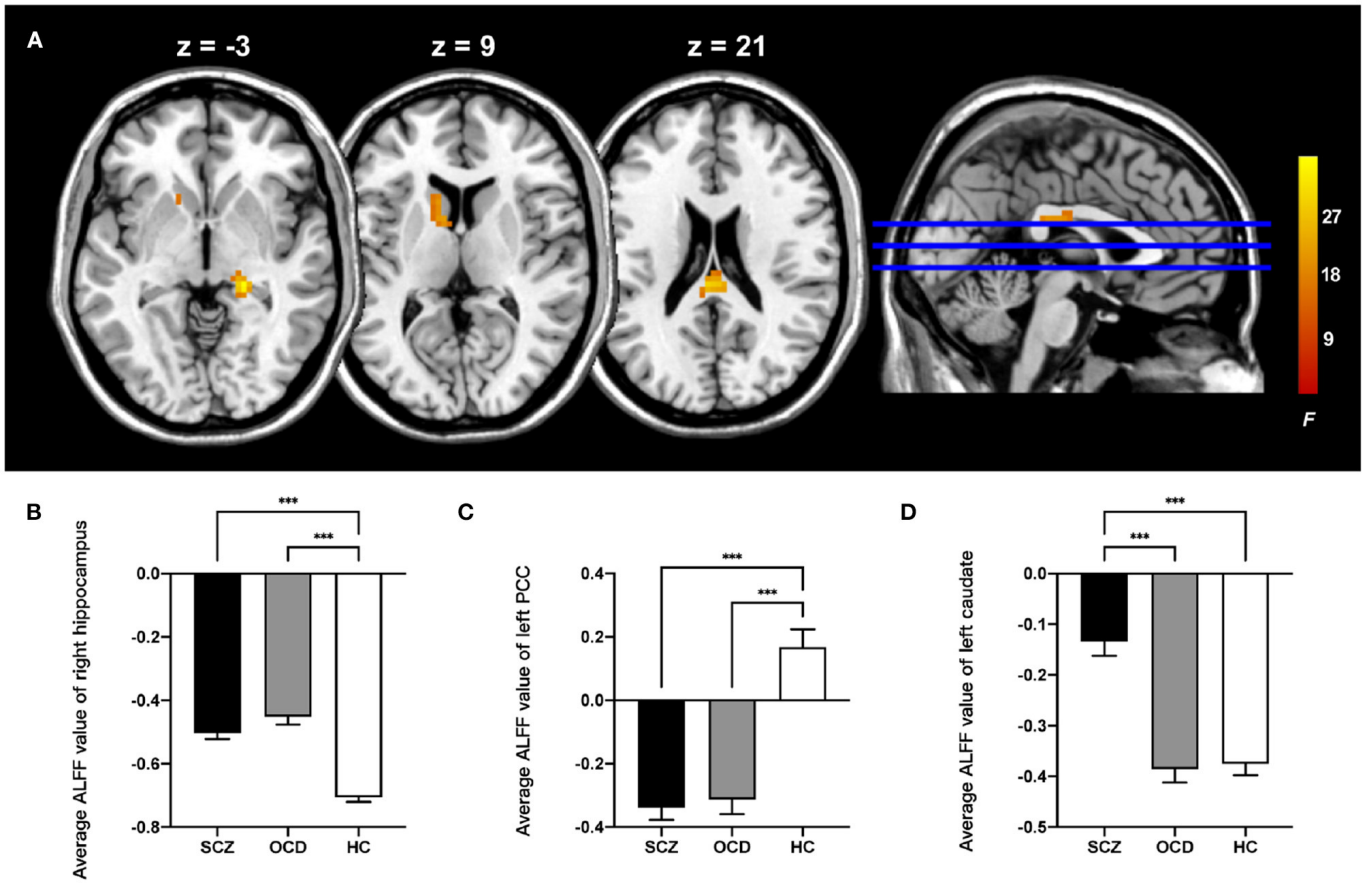
Comparison of ALFF in the patients with schizophrenia, obsessive-compulsive disorder, and healthy control subjects. **(A)** The significant brain region that showed significant difference among three groups (whole-brain voxel-level FWE corrected *P* < 0.05, *k* > 30). The bar graphs showed the averaged ALFF in the right hippocampus **(B)**, left posterior cingulate cortex **(C)**, and left caudate **(D)** within each group. PCC, posterior cingulate cortex; ALFF, amplitude of low-frequency fluctuations; FWE, family-wise error; ***P < 0.001, Bonferroni corrected.

For ALFF, three groups showed significant differences in the right hippocampus, left posterior cingulate cortex (PCC), and left caudate (whole-brain voxel-level FWE corrected *P* < 0.05, cluster size > 30, Table 2 and Figure 1A). We extracted the average value of ALFF in the above regions. Both the SCZ and OCD groups showed significantly increased ALFF values (the negative ALFF values decreased) in the right hippocampus and decreased ALFF in the left PCC than did HCs. The SCZ group showed significantly increased ALFF in the left caudate nucleus than did OCD and HC groups (*P* < 0.001, Bonferroni corrected, Figure 1).

**Table 2:** Results of ALFF analysis of the patients with schizophrenia, patients with obsessive-compulsive disorder, and healthy controls.

**Brain region**	**Hemisphere**	**Cluster size**	**MNI coordinates (x, y, z)**	**Peak *F* value**	**Voxel-level *P*_**FWE**_**
Hippocampus	Right	32	21, −30, −3	35.04	<0.001
Posterior cingulate cortex	Left	47	−3, −30, 21	28.88	<0.001
Caudate	Left	36	−9, 6, 9	21.57	<0.001
			−12, 15, 9	20.67	<0.001
			−15, 18, −3	15.93	0.023

*ALFF, amplitude of low-frequency fluctuations; MNI, Montreal Neurological Institute*.

**Figure 2:**
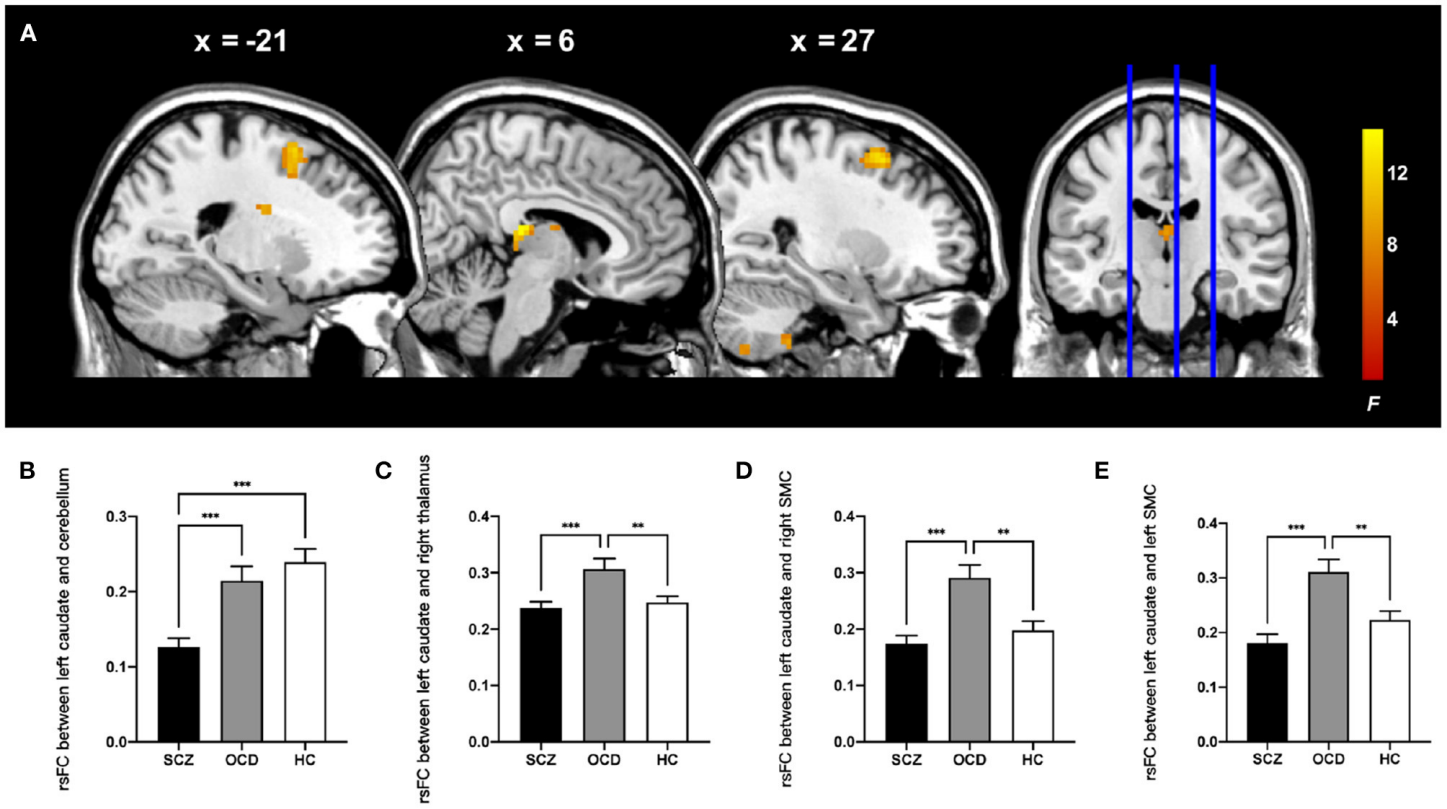
Comparison of ALFF-based rsFC analysis with the left caudate as the seed in the patients with schizophrenia, patients with obsessive-compulsive disorder, and healthy control subjects. **(A)** The significant brain region that showed significant difference among three groups (whole-brain cluster-level FWE corrected *P* < 0.05, *k* > 80). The bar graphs showed the averaged rsFC between the left caudate and right cerebellum posterior lobe **(B)**, right thalamus **(C)**, right SMC **(D)**, and left SMC **(E)** within each group. rsFC, resting-state functional connectivity; SMC, supplementary motor complex; ALFF, amplitude of low-frequency fluctuations. ***P < 0.001, **P < 0.01, Bonferroni corrected.

Then, by using the significant cluster within the left caudate as a seed, we found that rsFC between the left caudate, right thalamus, right cerebellum posterior lobe, and bilateral supplementary motor complex (SMC) including the supplementary motor area (SMA), supplementary eye fields (SEFs), and pre-SMA were significantly different among the three groups (whole-brain cluster-level FWE corrected *P* < 0.05, cluster size > 80, Table 3 and Figure 2A). The SCZ group showed significantly decreased rsFC between the left caudate and right cerebellum posterior lobe than both the OCD group and HCs. The OCD group showed increased rsFC between the left caudate and right thalamus, the left caudate, and the bilateral SMC than did both the SCZ group and HCs (*P* < 0.001, Bonferroni corrected, Figure 2).

**Table 3:** Results of ALFF-based rsFC analysis with the left caudate as the seed in the patients with schizophrenia, patients with obsessive-compulsive disorder, and healthy controls.

**Brain region**	**Hemisphere**	**Cluster size**	**MNI coordinates (x, y, z)**	**Peak *F* value**	**Cluster-level *P*_**FWE**_**
Thalamus	Right	102	6, −27, 12	14.49	0.003
Supplementary motor complex	Right	107	27, 15, 54	12.99	0.003
Supplementary motor complex	Left	103	−21, 12, 60	12.17	0.003
Cerebellum posterior lobe	Right	92	21, −42, −51	12.74	0.006

*ALFF, amplitude of low-frequency fluctuations; rsFC, resting-state functional connectivity; MNI, Montreal Neurological Institute*.

We also explored the clinical correlations of these neuroimaging alterations by computing the Pearson correlation coefficient between the average ALFF and rsFC values and the scores of symptom severity in the patients, with age and gender as covariates using Bonferroni correction. For the patients with SCZ, the rsFC between the left caudate nucleus and right cerebellum was positively correlated with the PANSS positive symptom score (*r* = 0.277, *P* = 0.010).

## Discussion

In this study, we compared resting-state activity changes using ALFF and seed-based rsFC in patients with SCZ, patients with OCD, and HCs. We found that (1) compared with HCs, both the patients with SCZ and OCD showed increased ALFF in the right hippocampus and decreased ALFF in the left PCC; (2) patients with SCZ exhibited increased ALFF in the left caudate than patients with OCD and HCs; (3) using the left caudate as a seed, patients with SCZ showed decreased rsFC between the left caudate and right cerebellum, which was correlated with the PANSS positive symptom score. Patients with OCD showed increased rsFC between the left caudate and right thalamus, the left caudate, and the bilateral SMC. Our results suggested that SCZ and OCD have common and distinct patterns of resting-state activity. Both of them exhibited abnormal ALFF in the hippocampus and PCC, while the striatum can reflect the differences. Patients with SCZ exhibited increased ALFF in the striatum and symptom-related weakened rsFC between the caudate and cerebellum. Enhanced rsFC of caudate–thalamus and caudate–SMC in OCD may be the important difference.

### Commonalities

#### Increased Amplitude of Low-Frequency Fluctuations in the Hippocampus

The altered ALFF values in the hippocampus across SCZ and OCD were consistent with previous ALFF studies that compared the two disorders with HCs (33, 34). The ALFF of hippocampus was increased in patients with SCZ (35) and associated with the severity of auditory and visual hallucinations (34). In patients with OCD, the ALFF of the hippocampus was also increased as compared with that of HCs, and the difference disappeared after 4-week treatment with the remission of the obsessive-compulsive symptoms (33). The hippocampus, which have been reported with both structural (reduced volume and thinner cortex) and functional abnormalities in SCZ and OCD (36, 37), might play a cardinal role in the neurobiology of both disorders through its effect on various cognitive and affective processes. Intrinsic hippocampal hyperactivity in the resting state is a characteristic feature of SCZ and related to cognitive dysfunction (38). OCD patients also exhibited cognitive impairments including attention, executive function, and memory (39). The similar higher ALFF in both SCZ and OCD represents the similar hyperactivity in the hippocampus, which may be related to the common impairment of cognitive function, especially the decline of memory ability.

#### Decreased Amplitude of Low-Frequency Fluctuations in the Posterior Cingulate Cortex

The PCC is highly connected to various brain regions with a high baseline metabolic rate, which is the core node of default mode network (DMN) but showed abnormal reduced glucose metabolism in SCZ (40). The ALFF and fALFF in the PCC showed a consistent decrease in the two low-frequency bands in SCZ and schizoaffective disorder (41). The fALFF of the posterior cortex (including the occipital lobe and the precuneus/PCC) was also reduced in SCZ (42). Similarly, DMN plays a key role in the pathophysiology of OCD, although there are relatively few reports on resting state dysfunction in the PCC. One study found that the network homogeneity of the PCC/precuneus of OCD was significantly reduced, which could be used as a candidate neuroimaging index to distinguish OCD from HCs (43). Our finding of reduced ALFF of the PCC in SCZ is consistent with previous studies. OCD patients performed a disassociation between the increased behaviors and correct appraisal on the need to make the action (44). SCZ patients have deviations in self-recognition. They both show decreased insight in varying degrees, which might be correlated with the abnormal function of DMN that is involved in internal emotional processing and self-referential directed thought (45).

### Distinctions

#### Increased Amplitude of Low-Frequency Fluctuations in the Dorsal Striatum in Schizophrenia

The ALFF of the striatum in patients with SCZ showed an increase that was specifically different from that in patients with OCD and HCs, which was similar to the findings of increased cerebral blood flow and glucose metabolism in the striatum in drug-naïve patients with SCZ (46) and consistent with the meta-analysis (24). The relationship between the striatum and SCZ is supported by the dopamine hypothesis of SCZ (18). Studies have found that dopamine-related striatal-thalamic-cortical rsFC in SCZ was abnormal in low-frequency oscillations, suggesting that the changes in dopaminergic function may lead to abnormal synchronization of neurons in subcortical circuits (47). It is proposed that the temporary retention of excessive spontaneous dopamine can temporarily combine with the striatal signaling pathway through stimulation, making irrelevant external or internal stimulation significance (18, 48). The dorsal striatum is usually involved in signaling threat-related information (49), which may explain why the delusions of SCZ patients in natural conditions are usually persecuted (18). Given another role of the dorsal striatum in the formation of habit (50) and the process of encoding stable value (51), it can be speculated that the dopaminergic dysfunction in the dorsal striatum accompanied with mental symptoms could aggravate the habit-oriented mode of cognition and rigid form of thought with unusual content (52). The increased ALFF in the dorsal striatum in SCZ may have relevance to the fact that the hallucinations and delusions of SCZ are not common in OCD.

#### Decreased Resting-State Functional Connectivity Between the Striatum and Cerebellum in Schizophrenia

Emerging human neuroimaging studies have discovered the existence of a large-scale cortex–striatum–thalamus–cerebellar functional loop. The cerebellum and striatum communicate with the thalamus and cortex through single and multiple synaptic connections (53) and may be sensitive to the disconnection of the whole brain in patients with SCZ, which is conceptualized as a synaptic signal communication that affects the nervous system. Ji et al. used a data-driven method to analyze the FC with the striatum and the cerebellum as independent seeds and found a high degree of similarity in the two whole-brain connection patterns in patients with SCZ with decreased rsFC between the striatum and the cerebellum (54), which is consistent with our results. The dysconnectivity in the cortico-striatal-thalamic-cerebellar pathway was strongly related to cognitive deficits (54). Dynamic stimulation of the cerebellum could affect the activities of multiple areas of the frontal cortex and effectively improve the cognitive ability of patients with SCZ (55). Given the role of the cerebellum in cognition (56), such as working memory (57), the weakened cerebellar rsFC of patients with SCZ might suggest the more severe cognitive impairment in SCZ than OCD. In addition, we observed a positive correlation between decreased caudate–cerebellar rsFC and the severity of positive symptoms, suggesting that the mild rsFC abnormalities may lead to the development of positive symptoms, whereas excessive abnormality might prevent the formation of positive symptoms (58).

#### Increased Resting-State Functional Connectivity Between the Striatum and Thalamus, Striatum and Supplementary Motor Complex in Obsessive-Compulsive Disorder

Previous studies suggested that OCD is related to abnormalities in the CSTC loop. The cerebral cortex projects the signal to the striatum, transmits the signal to the thalamus through the globus pallidus, and finally feeds back to the neuronal circuit of the cerebral cortex. Increased functional connectivity primarily within the CSTC circuits was observed in patients with OCD and their first-degree relatives (59). The SMC consists of the SMA, the SEFs, and the pre-SMA (60), which are important for movement preparation and behavioral sequencing. SMA send efferent neuro to the striatum directly and indirectly (60). Pre-SMA/SMA is also speculated to be related to the cause of impaired response inhibition with disability to inhibit irrelevant information and suppress responses to distractors in patients with OCD, which showed aberrant activations during working memory (61). The hyperactivity of pre-SMA during response inhibition was reported to be a candidate endophenotype of OCD (62). Furthermore, in OCD-relevant mouse model, M2 postsynaptic responses in the central striatum were significantly increased, which suggested that strengthened M2-striatal inputs might contribute in striatal hyperactivity and compulsive behaviors, where M2 is homologous to pre-SMA/SMA in human (63). SMA has also been identified as promising targets for repetitive transcranial magnetic stimulation to reduce OCD-related symptoms (64). The association of striatum and SMC may interfere with flexible transition between habitual and goal-directed behaviors, which act as impaired goal-directed behavior and more dependence on habitual behavior system, thus promoting the formation of stereotyped behavior and compulsive behavior in OCD (65). This characteristic is different from the deficit in goal-directed action in SCZ, which fails to integrate the causal knowledge of behavior outcome relationship with the change of outcome value to modify their action (66).

Consistent with our hypothesis, the striatum is the key brain region that showed abnormality in two diseases but present different patterns of lesions, which might be associated with different clinical features. This study still has several limitations. First, the sample size of the OCD group is relatively small, and the current study does not completely match between two patient groups. The findings of this study need to be verified in a more matched and larger sample. Second, we did not assess the obsessive-compulsive symptoms in patients with SCZ, and the PANSS was not measured in the OCD group, but the patients were recruited after strict SCID screening to confirm that there was no comorbidity. The assessment of symptoms can be added in the further study to confirm the validity of our results. Third, many patients in our study were medicated before recruitment, but due to the use of different types of psychotropic medications (antipsychotics and antidepressant medications), we could not add the equivalent dosages as covariates in the statistical analysis. Future work can be carried out in un-medicated patients. Finally, it requires more experimental evidence to support the clinical application of our findings.

In summary, the hippocampus and PCC are common regions presenting abnormal local spontaneous neuronal activities in both SCZ and OCD, while the abnormality of the striatum can reflect the differences. Increased ALFF in the striatum and symptom-related weakened rsFC between the caudate and cerebellum showed SCZ specificity. Enhanced rsFC between the caudate and SMC in OCD may be a key characteristic in OCD. Our research shows the similarities and differences between the two diseases from the perspective of resting-state fMRI, which provides clues to understand the disease and find methods for treatment.

## Data Availability Statement

The raw data supporting the conclusions of this article will be made available by the authors, without undue reservation.

## Ethics Statement

The studies involving human participants were reviewed and approved by the ethics committee of Peking University Sixth Hospital. The patients/participants provided their written informed consent to participate in this study.

## Author Contributions

YZ completed the data analysis, wrote the first draft of this manuscript, and edited the subsequent versions. YZ, JL, QL, and LL are responsible for the data collection. JL, XZ, HY, and WY gave critical revision for the manuscript. JY, DZ, HY, and WY were responsible for the designing the study. All authors have read and approved the final version of this article. We thank the National Center for Protein Sciences at Peking University in Beijing, China, for assistance with MRI data acquisition.

## Conflict of Interest

The authors declare that the research was conducted in the absence of any commercial or financial relationships that could be construed as a potential conflict of interest.
